# Women with large breasts are at an increased risk of advanced breast cancer

**DOI:** 10.1186/1477-7800-5-16

**Published:** 2008-06-30

**Authors:** Chaminda Sellahewa, Peter Nightingale, Amtul R Carmichael

**Affiliations:** 1 Department of Surgery, Russells Hall Hospital, Dudley, UK; 2 Wolfson Computer Laboratories, University Hospital Birmingham NHS Foundation Trust, Birmingham, UK; 3 Department of Surgery, Russells Hall Hospital, Dudley, UK

## Abstract

**Background:**

The risk of nodal metastasis is higher in women with bigger breast. It is not clear if this increase is due to the size of the breast (largely related to obesity) or is the result of larger tumour size at presentation (due to delayed diagnosis). It is hypothesised that women with large breasts are more likely to have node positive disease mainly attributable to their breast size.

**Patients and methods:**

One hundred and twenty consecutive patients who underwent mastectomy during the year 2004 and 2005 for primary breast cancers in a large Teaching Hospital were included in the study. Patient's variable and tumour variable were collected and analysed by SPSS^® ^computer programme.

**Results:**

It was found that big breasted women (those patients with mastectomy weight greater than 800 g) had a significantly greater tumour size than those with smaller breasts (p = 0.019, Mann-Whitney test) but there was no significant difference in grade (Kendall's tau-b = 0.055, p = 0.57) or lymph node positivity (Kendall's tau-b = 0.011, p = 0.93) between the two groups. Although, the tumour size was significantly greater in those with lymph node metastases (p < 0.001) but mastectomy weight was not found to be significantly greater in those with lymph node metastases (p = 0.11). For patients with similar tumour sizes mastectomy weight was not significantly greater in those patients with lymph node metastases (p = 0.28).

**Conclusion:**

It is concluded that increased incidence of lymph node positivity at presentation big-breasted women is because of larger size of the primary tumour and not due to the size of the breast alone.

## Background

The present literature on the large size of breast and the stage of breast cancer is controversial. The larger size of the breasts is more common in obesity which is associated with poor prognosis of breast cancer in both pre and postmenopausal women[1]. It is argued that women with large breasts have more advanced stage of disease at presentation as the larger breasts of obese women may make it difficult to detect early disease by palpation. It is also suggested that women with large breasts may develop lymph node metastasis at a smaller size of primary breast cancer than those with smaller breast because of altered host responses [2]. This poor outcome of breast cancer in obesity can be attributed to either the larger tumour size at presentation or the increased susceptibility to lymph node positivity. The evidence for either of these causes of poor prognosis is complex and controversial. Most of studies addressing these issues are case control or cohort studies, the size of a large breast has not been defined by most studies and the methodology for measuring big breast is also varied using mammographic volume measurements, self-reported bra cup size and the size of mastectomy specimen [3-14]. Most but not all studies have found an increasing tumour size with larger breasts but only a few reported studies have found a high rate of node positivity in larger breasts. It was hypothesised that women with large breasts are more likely to have node positive disease mainly attributable to their breast size.

## Patients and methods

One hundred and twenty primary breast cancers operated at a large Teaching Hospital were included in the study. These were consecutive patients who underwent mastectomy during the year 2004 and 2005. Patient's variable and tumour variable were collected and analysed by SPSS computer programme.

### Statistical methods

Spearman correlation was used to measure association between continuous variables, the Mann Whitney test for the comparison of continuous variables between two groups and Kendall's tau-b statistic to analyse ordinal variables such as grade and lymph node positivity. Binary logistic regression analysis was used to determine whether, for patients with similar tumour sizes, mastectomy specimen weight was a significant predictor of the presence of lymph node metastases.

## Results

A large breast was defined as a breast with mastectomy specimen weight of 800 grams or more. It was found that increasing weight of the mastectomy specimen was associated with increasing risk of large tumour (rho = 0.26, p = 0.008). The tumour size was significantly greater (p = 0.019, Mann-Whitney test) in those with mastectomy weight greater than 800 g (Table 1). There was no significant difference in grade (Kendall's tau-b = 0.055, p = 0.57) or lymph node positivity (Kendall's tau-b = 0.011, p = 0.93) Table 1. The primary tumour size was significantly greater in those patients with axillary lymph node metastases (p < 0.001) but the mastectomy weight was not found to be significantly greater in those with lymph node metastases (p = 0.11). For patients with similar tumour sizes mastectomy weight was not significantly greater in those with lymph node metastases (p = 0.28) Table 1.

**Table 1:** The relationship between mastectomy weight and lymph node positivity

	**Mastectomy ** **weight < 800**	**Mastectomy weight equal to or** **more than 800**	**P value and test**
Tumour size in mm	21 (15–36.25)	27 (19.5–52.5)	P = 0.019, Mann-Whitney test
Grade			
I	7 (11%)	5 (10%)	Kendall's tau-b = 0.055, p = 0.57
II	37 (58%)	25 (52%)	
III	20 (31%)	18 (38%)	
Lymph node			
Negative	17 (27%)	9 (20%)	Kendall's tau-b = 0.011, p = 0.93
1–3	14 (22%)	14 (31%)	
>3	33 (52%)	22 (49%)	

Obese women were more likely to have node positive disease but on Binary logistic regression it was found that mastectomy weight itself was not a significant predictor of lymph node positivity p = 0.69, while taking variable such as tumour size, grade and ER status into account.

## Discussion

This study demonstrates breast size bigger than 800 grams is associated with increased risk of larger tumours and large breast size alone is not associated with increased incidence to lymph node metastasis. Therefore, the advance disease at presentation of breast cancer is attributable to the size of the primary breast cancer. In the present study the lymph node positivity rate in the large and non-large breasts were similar suggesting the nodal metastases is determined by the size of the primary tumour and not by the size of the breast. There are other factors that contribute to lymph node positivity. The most significant of which include age of the patient, grade of the tumour and oestrogen receptor status. The present study found that there was no statistically significant difference between theses factors and lymph node positivity in women with large breast and small breast.

### Breast size and lymph node positivity

The present study did not find any evidence that the rate of lymph node positivity was higher in women with larger breast. The present study does not concord with an earlier study, which reported that patients with big breasts had more involved lymph nodes than women with small breasts, whether breast size was assessed by weight or thickness [15]. We used mastectomy weight as a measurement of breast size because this is the most definite, unbiased and reproducible measure of the size of the breast. This can also be regarded as a weakness of our study because women who with very large breast who underwent breast-conserving surgery might not be included in this cohort. As women with highest quartile of the breast weight may have undergone breast-conserving surgery, this may have diluted the effect of size and the nodal metastasis in the study population.

### Mammographic breast volume and breast cancer size

In a study of 250 women breast volume calculated from mammograms and compared with those of 250 age-matched controls found that breast cancer patients had larger breasts than age-matched healthy women [16]. Data for 2325 cases and 7008 controls from 4 centres found that the larger cup size was associated with an increased risk of breast cancer (P about 0.026), although the association was found only among postmenopausal women [6]. The influence of breast size (measured by volume from mammography) on the prognosis of 196 patients with early breast reported that breast size was significantly associated independently with T stage (z = -1.91, P = 0.05). Even though women with larger tumours at presentation had larger breasts, breast size was not a significant prognostic factor in early breast cancer [17].

### Bra cup size and breast cancer size

A prospective study of 123 patients with primary breast cancer to determine whether patients with larger breasts (determined by cup size) have larger tumours reported that patients with bigger breasts had larger tumours in both symptomatic and screen detected group [18]. In a large, population-based case-control study of women aged 50 to 79 years in the USA Breast size before a pregnancy was found to be a positive predictor of postmenopausal breast cancer, but this association was limited to those who were especially lean as young women [19]. The Nurses' Health Study II reported that a larger bra cup size at a young age is associated with a higher incidence of premenopausal breast cancer, though this association is limited to leaner women after studying the Bra cup size at age 20 among 89,268 premenopausal women aged 29–47 [20]. A case-case comparison found that Odds of late-stage disease were increased with larger bra cup size (OR for cup D vs. cup A = 1.61, 95% CI 1.04–2.48). These relationships were not modified by the method of detection. Authors concluded that differences in etiologic effects, rather than differences in detection methods, might explain the relations observed between stage and both BMI and breast size [21].

## Conclusion

It is concluded that Increased lymph node positivity at presentation of breast cancer in big-breasted women is because of larger size of the primary tumour and not due to the size of the breast alone.

## Authors' contributions

ARC conceived the idea and coordinated the data collection and analyses, participated in all stages of the study and drafted the manuscript, CS collected the data and PN participated in the performed the statistical analysis. All authors read and approved the final manuscript.

## References

[B1] Carmichael AR (2006). Obesity as a risk factor for development and poor prognosis of breast cancer. BJOG.

[B2] Daniell HW (1988). Increased lymph node metastases at mastectomy for breast cancer associated with host obesity, cigarette smoking, age, and large tumor size. Cancer.

[B3] Carmichael AR (2006). Obesity as a risk factor for development and poor prognosis of breast cancer. BJOG.

[B4] Kusano AS, Trichopoulos D, Terry KL, Chen WY, Willett WC, Michels KB (2006). A prospective study of breast size and premenopausal breast cancer incidence. Int J Cancer.

[B5] Ingram DM, Huang HY, Catchpole BN, Roberts A (1989). Do big breasts disadvantage women with breast cancer?. Aust N Z J Surg.

[B6] Hsieh CC, Trichopoulos D (1991). Breast size, handedness and breast cancer risk. Eur J Cancer.

[B7] Hoe AL, Mullee MA, Royle GT, Guyer PB, Taylor I (1993). Breast size and prognosis in early breast cancer. Ann R Coll Surg Engl.

[B8] Hasenburg A, Grothey A, Jaspers V, Gitsch G, Spatling L (2000). Breast size as risk factor for tumor size at diagnosis. Anticancer Res.

[B9] Hall HI, Coates RJ, Uhler RJ, Brinton LA, Gammon MD, Brogan D (1999). Stage of breast cancer in relation to body mass index and bra cup size. Int J Cancer.

[B10] Hall IJ, Newman B, Millikan RC, Moorman PG (2000). Body size and breast cancer risk in black women and white women: the Carolina Breast Cancer Study. Am J Epidemiol.

[B11] Egan KM, Newcomb PA, Titus-Ernstoff L, Trentham-Dietz A, Baron JA, Willett WC (1999). The relation of breast size to breast cancer risk in postmenopausal women (United States). Cancer Causes Control.

[B12] Duffett RH (1993). Breast size and prognosis in early breast cancer. Ann R Coll Surg Engl.

[B13] den TI, Peeters PH, van Noord PA (2004). Increase in breast size after menopause: prevalence and determinants. Maturitas.

[B14] Daniell HW (1988). Increased lymph node metastases at mastectomy for breast cancer associated with host obesity, cigarette smoking, age, and large tumor size. Cancer.

[B15] Ingram DM, Huang HY, Catchpole BN, Roberts A (1989). Do big breasts disadvantage women with breast cancer?. Aust N Z J Surg.

[B16] Scutt D, Manning JT, Whitehouse GH, Leinster SJ, Massey CP (1997). The relationship between breast asymmetry, breast size and the occurrence of breast cancer. Br J Radiol.

[B17] Hoe AL, Mullee MA, Royle GT, Guyer PB, Taylor I (1993). Breast size and prognosis in early breast cancer. Ann R Coll Surg Engl.

[B18] Hasenburg A, Grothey A, Jaspers V, Gitsch G, Spatling L (2000). Breast size as risk factor for tumor size at diagnosis. Anticancer Res.

[B19] Egan KM, Newcomb PA, Titus-Ernstoff L, Trentham-Dietz A, Baron JA, Willett WC (1999). The relation of breast size to breast cancer risk in postmenopausal women (United States). Cancer Causes Control.

[B20] Kusano AS, Trichopoulos D, Terry KL, Chen WY, Willett WC, Michels KB (2006). A prospective study of breast size and premenopausal breast cancer incidence. Int J Cancer.

[B21] Hall HI, Coates RJ, Uhler RJ, Brinton LA, Gammon MD, Brogan D (1999). Stage of breast cancer in relation to body mass index and bra cup size. Int J Cancer.

